# Treatment for Sea Sickness—Liverpool Hospitals—School for Nurses—English Country Surgeons, Manner of Conducting Business—London—New Anæsthetic in Use—Dangers of Chloroform

**Published:** 1867-08

**Authors:** Edmund Andrews

**Affiliations:** 23 Cecil Street, Strand, London


### TREATMENT FOR SEA-SICKNESS—LIVERPOOL HOSPITALS—SCHOOL FOR NURSES—ENGLISH COUNTRY SURGEONS, MANNER OF CONDUCTING BUSINESS—LONDON—NEW ANÆSTHETIC IN USE—DANGERS OF CHLOROFORM.

LONDON, July 5th, 1867.

Conformably to my promise, I drop you a few lines respecting my observations upon this side of the water. I took the steamer *City of Paris*, for Liverpool, on the 15th ult. As the weather proved calm, I had no opportunity to make discoveries on that soul and stomach stirring subject, sea-sickness. By conversing with the ship's surgeons and others, I judge that all the numerous remedies for this nuisance, so far as they have any beneficial effect, may be reduced to two classes:—1st. Stimulants to the mucous membrane of the stomach, and to the nervous system. Essential oils, chloroform taken internally, brandy, aromatics, and other irritants seem to occupy the attention of the nervous system somewhat, and have a moderate tendency to obviate the nausea. A strong mental impression has the same effect.

2d. Cathartics, which act on the portal circulation, are very positive in their influence. Persons very subject to sea-sickness often take a voyage in entire comfort by the following precaution:—Take ten grains of blue mass the night before embarking; follow it the next morning with a brisk cathartic of Seidlitz powders. A voyage at sea is almost always constipating in its effects, and a repetition of the medicine once or twice on the passage may be necessary.

On my arrival at Liverpool, I spent a few days in examining the hospitals of that city. There are three general hospitals, all supported by private charity. In addition to these, there is a dispensary, and a large hospital in the workhouse. These institutions are attended by skilful men, and are a great public benefit. I may say, however, that I have not seen, in Philadelphia, New York, Liverpool, or London, more than one well-ventilated hospital. The Middlesex Hospital, London, is the only one I have found where the surgical patients receive air at all approaching in purity that which I have always had for my patients in Mercy Hospital, Chicago.

In one of the Liverpool hospitals, there is a school for female nurses, who take a training for twelve months. They then compensate the institution by giving to it a certain portion of their wages for the next two years, towards the hospital fund.

On my way from Liverpool to London, I stopped at the beautiful, little old town of Warwick. We have here Warwick Castle, in complete repair, with its great hall full of old armor and other relics. Near by, are the ruins of Kenilworth Castle, the scene of Scott's novel, "Kenilworth," as well as Stratford-upon-Avon, Shakespeare's birthplace, and numerous other spots of antique interest. I was here most hospitably entertained by Surgeon NUNN and his family. Mr. NUNN (surgeons are called Mr. in England) is in a very active and successful practice in and around Warwick, where he stands deservedly high in public esteem. It may not be known to all your readers that the surgeons in small English towns are general practitioners, though called surgeons. As a rule, they ride about, visiting patients of every kind, like an American physician, but they do not have to ride so far, and I am gratified to say that they have much better roads to go on than we. The surgeon keeps a stock of medicines in his house, and having seen his patient, returns and sends the medicine by a messenger. It is difficult to compare their rate of compensation with ours, on account of the manner in which it is collected. As a general rule, there is no charge made for the visits, but the whole expense is put upon the medicines. The patient is charged a certain rate for every two pills, so much a-piece for powders, and so much for a draught, etc. As a class, the country surgeons are well trained and perfectly competent.

A new anæsthetic, or rather a new attention to one previously proposed, is being pressed upon the profession in London, by Dr. PROTHEROE SMITH, of the Hospital for Women, and, from the experiments thus far made, there is reason to hope that it may combine the energy of chloroform with the safety of ether, besides being much more agreeable to inhale than either of them. Its production in a crude form is easy and cheap, but its complete purification is rather difficult. I subjoin Dr. SMITH's description of the method.

The tetrachloride, or, as it used to be called, the bichloride of carbon, [Sir James Simpson has concisely termed it "chlorocarbon,] is the highest of a series of chlorides of four grades, as follows:

| C=12 | C=6 |
|---|---|
| 1. Protochloride of carbon, $CCl_2$ | 1. Subchloride of carbon, $C_4Cl_2$ |
| 2. Dichloride of carbon, $C_2Cl_4$ | 2. Protochloride of carbon, $C_4Cl_4$ |
| 3. Trichloride of carbon, $C_2Cl_6$ | 3. Sesquichloride or perchloride of carbon, $C_4Cl_6$ |
| 4. Tetrachloride of carbon, $CCl_4$ | 4. Bichloride of carbon, $C_4Cl_4$ |

The subjoined table exhibits at a glance some of their physical properties:

| ...... | Usual Condition. | Boiling Poi't, C,......... | Freezing p't, C,......... | Specific gravity,......... | Sp. gr. of Vapor,......... | Soluble in Alcohol, Ether and Oils. | Insoluble in Water, Acids and Aqueous Alkalies,...... | Action on the Skin and Mucous Membrane. |
|---|---|---|---|---|---|---|---|---|
| Protochloride of carbon... | { Crystals. | ...... | ...... | ...... | ...... | In ether | ...... | ...... |
| Dichloride..... | Liquid. | 122° | Liquid —18° | 1·619 | 5·82 | In all. | Yes. | Taste first intensely sweet and pungent; afterwards bitter. |
| Trichloride... | †Crystalline | 182° | ...... | 2· | 8·157 | Ditto. | Ditto. | ...... |
| Tetrachloride | Liquid. | 77° | —27° | 1·56 | 5·3 | Ditto. | Ditto. | Very slight on mucous membrane; on skin none. |

†There has been obtained an isomeric liquid Trichloride, the vapor of which has a density of 4·082.

From this table it will be seen that the tetrachloride, from its superior volatility, is the only known chloride of carbon suited to administration by inhalation.

Of the numerous methods of preparing the tetrachloride of carbon, that devised at the Royal Institution by Messrs. Frankland and Duppa appears to be the simplest and most economical. The following is a brief outline of the process:

A Woulfe's bottle is filled to rather less than one-fourth of its capacity with bisulphide of carbon, to which has been added either a little sulphur, or, what is better, bichloride of sulphur; one per cent. of either of the above substances will suffice. A stream of perfectly dry chlorine is now passed through the mixture, and continued until no further absorption takes place; this occurs when the liquid has increased to about four times its original volume. As the liquid in the bottle becomes very hot during the process, means for keeping it cool must be resorted to.

The brownish red liquid produced now consists of tetrachloride of carbon and tetrachloride of sulphur saturated with uncombined chlorine. Were water simply added to this liquid a large deposit of sulphur would take place, hydrochloric acid, sulphurous acid, and sulphuretted hydrogen being procured at the expense of the tetrachloride of sulphur, and a large quantity of tetrachloride of carbon set free; but as tetrachloride of sulphur, when brought into contact with bisulphide of carbon, is found to yield tetrachloride of carbon and bichloride of sulpur, according to the subjoined equation:

$$4\,S\,Cl_4 \quad + \quad C\,S_2 \quad = \quad C\,Cl_4 \quad + \quad 6\,S\,Cl_2$$

| Tetrachloride of Sulphur | Bisulphide of Carbon | Tetrachloride of Carbon. | Dichloride of Sulphur. |
|---|---|---|---|

advantage of this reaction is taken, and a certain quantity of bisulphide in small portions is gradually added, and the whole submitted to distillation. After three or four rectifications the liquid separates into two portions, one consisting of tetrachloride of carbon, containing a little bichloride of sulphur and the other of bichloride of sulpur containing a little tetrachloride of carbon. The impure tetrachloride is now treated with water and milk of lime distilled, when the substance nearly pure distils over with a little water: a rectification or two *per se* will suffice to obtain it in a state of absolute purity.

Messrs. Hopkin and Williams, of New Cavendish-street, who have succeeded in producing a very pure specimen of the tetrachloride of carbon according to the above-described process, have found that, before its final washing, the addition of a little ammonia very much improves its character, and helps to deprive it of the last traces of bisulphide of carbon and chloride of sulphur.

A specimen having been obtained from the laboratory of the Royal Institution, about half a drachm on a handkerchief was inhaled. Its vapor was found to be agreeable, having a delicate perfume not unlike that of quince, and imparting at first a sensation of coolness to the throat, similar to that experienced in drawing in one's breath after taking peppermint, followed by a feeling of warmth on the surface of the body generally. This was succeeded by a feeling of calmness and freedom from the exhaustion which had previously been felt, and which did not return during the remainder of the day; and sleep that night was more sound than usual. The experiment was repeated on the following day, with similar results. On March 23d, having obtained a perfectly pure specimen of the fluid, twenty minims on a handkerchief were inhaled, repeating the dose when it had evaporated. Its anæsthetic effects were very rapid, preceded by an agreeable sense of drowsiness &c., and other sensations similar to those which I have experienced from chloroform, but in a less degree, and giving place in about two minutes to calm sleep, after which scarcely a minute elapsed before the return of complete consciousness. Sleep was again calm and undisturbed through the succeeding night. With the assistance of Dr. Heywood Smith its effect on insects and some of the lower animals was tried. In the case of insects it was found that whereas chloroform, when dropped on their heads, was quickly fatal, they soon recovered when the tetrachloride in like manner was employed.

Dr. Smith has now given this article about one hundred times. His conclusions are these:—It is a powerful anæsthetic, but when pure does not sicken the stomach nor excite cough in administration; it is more prompt than ether, but slower than chloroform in its action. In the inferior animals he has pushed it to a fatal result, in order to observe the effects. From his observations, he concludes that, while chloroform is apt to cause death in a sudden and unexpected manner, giving no opportunity for precaution, the tetrachloride of carbon, when pushed to fatal extent, brings on its results gradually, giving opportunity to regulate the quantity and avoid the danger. He finds it extremely useful in the Hospital for Women, as an anodyne for headaches, and as a sedative to hysterical excitability.

In my opinion, this article promises to become of great value, but further experience is necessary, in order to test the question of its relative safety. Its delicate, fruity odor, and its un-irritating, un-nauseating character are great advantages, if it shall prove to have the safety of sulphuric ether. Dr. Smith gives it in an inhaler instead of upon a napkin.

The English surgeons universally use chloroform instead of sulphuric ether in their operations. They keep no statistics of the results, but when I question them upon the point, they generally say that deaths occasionally occur, but so rarely as to be of little consequence. I have, however, carefully pushed my inquiries among the officers of the various hospitals, and I am of the opinion that they seldom exceed two thousand administrations of chloroform without a death occurring. I am carefully gathering statistics upon this point, and shall have something further to say about it in a future letter.

Yours very truly, EDMUND ANDREWS, M.D.

*23 Cecil Street, Strand, London.*

